# Disposal of the Fæcal and Other Refuse of London on Rational Principles

**Published:** 1857-04

**Authors:** Philip B. Ayres

**Affiliations:** late Lecturer on Materia Medica at the Grosvenor Place School of Medicine.


### ORIGINAL COMMUNICATIONS.

### ON THE DISPOSAL OF THE FÆCAL AND OTHER REFUSE OF LONDON ON RATIONAL PRINCIPLES, IN CONTRAST WITH THE SCHEMES OF SEWERAGE NOW BEFORE THE PUBLIC.

By PHILIP B. AYRES, M.D.Lond., late Lecturer on Materia Medica at the Grosvenor Place School of Medicine.

In a memorial presented nine years since to the Metropolitan Sanitary Commission, after describing what was then known of the chemical phenomena of putrefactive fermentation, I made the following observations: "If we take the solid excrement at four ounces *per diem* for each individual, it will give for the entire population of London 223 tons of fæcal matter daily, or 81,395 tons annually. The solid dry matter of fæces amounts, according to the analysis of Berzelius, to about one-fourth of the whole, which would give 56 tons of dry matter *per diem*, or 20,440 tons; or, calculating it at one-fifth, which would probably be nearer the truth, 44 tons daily, or 16,239 annually." Adding to this at least "30 tons of the dry matter of the urine, we have 46,500 tons of dry matter, capable of putrefaction on exposure to the air, to be thrown into the Thames each year; an amount approximating to one thousand tons weekly, in addition to a very large quantity of other substances derived from a variety of trades and manufactures, the putrid blood and fluids of slaughter-houses, etc. At the same time, a large quantity of paper impregnated with putrefiable matter, and the liquid refuse of kitchens, will find its way into the sewers, increasing the mass of putrescible matter to an enormous extent; and all these substances being mixed with the silt (or mud), especially the viscid matter of the fæces, will be more or less deposited on the strand of the Thames. The Thames, being a tidal river for some distance above London, is not uniform in its flow like other rivers; each return of the tide reverses the current, and will tend to wash the putrid mud backwards and forwards, instead of at once carrying it away. When at low water, many acres of putrid matter will be freely exposed to the air, and putrid emanations will rise in abundance in hot weather, when these are most dangerous. A little consideration will shew, that by carrying the whole of the night soil and other drainage into the Thames, a greater nuisance will be produced than is now caused by the imperfect cesspools, which have been stigmatised as *barbarous.*"

These remarks were penned before the gigantic schemes of sewerage, now so well known, were promulgated; but it is evident that they apply to every part of the Thames, from London to the sea, and it is manifest that in process of time such an accumulation of fœtid mud must occur on the strand of the Thames for miles above and below the embouchure of the sewers, as to render that portion positively pestiferous to the inhabitants of its banks and the floating population of the river; and to absolutely forbid all schemes involving the *complete* drainage of the metropolis into the Thames. On these grounds it is not so much the question how far the refluent tide will carry upwards the sewerage, as the condition of the strand of the Thames, ten, twenty, or fifty years after the entire drainage has been cast into any part of the river. In all the notices and objections to these gigantic schemes of sewerage I have never seen any allusion to this, perhaps the strongest of objections to permitting the entire fæcal matters of London being thrust into the Thames; but I believe it impossible to deny that such must be the eventual result, should any scheme, involving discharge of all the London filth into the river, be adopted.

Another objection, to my mind of considerable force, is, that by making sewers of such enormous magnitude and length, which can never be full, and by discharging into these the whole of the fæcal matters, so many "elongated cesspools" will be constructed, in which all the conditions favourable to rapid putrefaction—movement, and intermixture with atmospheric air, for example—are present; and such a plan will result in a tenfold greater nuisance than has hitherto existed in the old system of cesspools and drainage; for it is undeniable that no system of trapping can render such sewers impermeable, and the mephitic gases and vapours will find their way into the surrounding atmosphere, in spite of all precautions that may be taken to prevent such an occurrence. These objections are, I apprehend, fatal to the system of sewerage at present proposed, which, if carried out, will entail an expense of millions on the metropolis, enormously increase the parochial rates, and end in disappointment and vexation.

It is a curious fact that, among a people deeming themselves eminently *practical* and priding themselves especially on this mental quality, statements of the wildest character, based only on theoretical imaginings, should have been permitted to obtain absolute sway over the public mind, and that the first step in inductive philosophy should have been entirely ignored—that of ascertaining, by careful and well directed experimental inquiry, the physical and chemical properties of the matter in question. Where, may I ask, are the results of such inquiries to be found? Certainly not in the records of the Sanitary Commission, nor of the subsequent Boards of Health, with the single exception that some members of the original Board, and their ardent supporters, duly attended the opening and emptying of certain cesspools, and denounced them at once as "abominations," because, during the process, a considerable stench was created. Surely such crude experiments, if I may so desecrate the term, cannot be cited as instances of the exertion of philosophical acumen, or as adding one particle to our already existing stock of knowledge on the subject.

To illustrate further the total absence of scientific inquiry as to the emanations constantly arising from putrescent matter, Dr. Southwood Smith and the sanitarians most positively affirmed that the constituent of these mephitic exhalations, which produced disease, was sulphuretted hydrogen, until I demonstrated* by experiments that, in the first place, only a minute proportion of this gas is present in the most fœtid of these emanations; and in the second, that persons may positively inhabit an atmosphere charged with a far larger proportion of this gas without suffering from the diseases attributed to the action of sulphuretted hydrogen. I may further affirm, from extensive research, and without fear of contradiction, that there is probably no one subject within the range of chemical inquiry on which less is known, than the subject at present under consideration—the phenomena of putrefaction of animal and vegetable substances. It is quite true that we are acquainted with several of the chief gaseous products of putrefaction—ammonia, and carburetted, phosphuretted, and sulphuretted hydrogen, for example; but, in addition to these, there are a number of less well defined substances, on which absolutely nothing has been ascertained, and these are indubitably the most important.

It is an almost universal opinion, that fæcal matters putrefy with greater facility than other animal matters, and that cesspools in which such matters are accumulated are foci of the putrefactive fermentation; and that therefore immense quantities of mephitic gases, highly injurious to the community, are produced, and must be got rid of as soon as possible. Never was a more mischievous or a more erroneous idea promulgated. I am prepared to demonstrate experimentally, in the most absolute manner, to any scientific commission, empowered to examine the subject, that there *is no putrefactive fermentation in the mass of the matter contained in a cesspool, that the small amount of putrefactive fermentation that does occur in a cesspool is con-*

* *Lancet.*

*fined to the surface of the matter, and that but a very minute quantity of sulphuretted hydrogen is generated under these circumstances.* I challenge the investigation; and I am confident, from the results of repeated and careful experiment, that the startling announcement I have now made will be proved to be correct.

In the beneficent plan of creation, such conditions have been imposed on animal matter, that when deprived of life it shall be as little injurious as possible to living beings; it either forms food for other animals or plants, or, if not adapted for these purposes, it is endowed with peculiar properties which retard its decomposition, and thus admit of such great dilution of its emanations with atmospheric air as to render them practically innoxious. The human fæces, less adapted than those of other animals as food for insects, possess a peculiar property, by which putrefactive change is greatly retarded. When fresh fæces, or night soil, remain at rest, the surface in the course of a few hours becomes covered by a tough film, by which access of atmospheric air is almost wholly prevented, and putrefactive change so much retarded that scarcely any odour is emitted; and such is the case in certain places in the neighbourhood of London (Bow Common, for example), where large pools of night soil, from one to two feet in depth, and covering a large extent of surface, are exposed to the air for the manufacture of manure. When these large pools are allowed to remain quiescent, the film, of which I have spoken, forms on the surface, and far less odour is emitted than from neighbouring chemical works; but if the night soil be stirred by raking, and thus intermingled with atmospheric air, and a fresh surface exposed, a fearful odour is emitted, until, after a few hours, the film has again formed on the surface. After a while, if the matter be left undisturbed, it becomes covered with minute fungi, or mouldiness, which feed on the animal matter, altogether change its character and odour, and ultimately reduce it to imputrescible and innocuous matter, or disperse it in the form of carbonic acid and ammonia through the atmosphere.

When investigating this subject, nine years ago, I half filled a wide-mouthed glass vessel with night soil. After the first fermentation and extraction of gases, consequent on admixture with atmospheric air in the necessary manipulation, was over, the film I have described formed on the surface. No bubbles of gas could be seen, scarcely any odour was emitted, and the surface became covered with mouldiness. This vessel remained standing uncovered in my laboratory for more than a year; but even during the heat of summer no offensive odour was perceptible in the room, and scarcely a trace of odour could be perceived on approaching the nose to the vessel; but when the mass was afterwards disturbed, putrefaction recommenced, and the offensive odour was renewed with pristine force.

Very recently a quantity of mixed fæces and urine, from a patient suffering from typhus, was placed in a common test glass, holding nearly half a pint, and closely covered by an evaporating dish. This liquid matter very soon, in like manner, became covered by a film, and the offensive odour, at first very strong, almost entirely ceased to be evolved, and not a single bubble of gas escaped from the fluid during several months that it remained standing in my laboratory. A small quantity of sulphuretted hydrogen was evolved, which darkened the lead contained in the glass. It is a curious feature in the putrefaction of purely animal substances, as urine, fibrin, albumen, etc., that scarcely any permanent gases are evolved; and these substances undergo the full putrefactive changes in closed vessels, partially filled with air, without breaking the vessel or forcing out the stopper; while vegetable matters, or a mixture of vegetable with animal matters, such as night soil, emit large quantities of uncondensable gases during the active putrefaction, which occurs in consequence of free admixture with atmospheric air. When, for example, night soil is agitated with water and air, in a half-filled bottle, and the bottle closely corked, very active putrefaction ensues, and so large a quantity of gas is evolved, that the bottle bursts or the cork is driven forcibly out, and the liquid, expanded to many times its original bulk, flows over the sides of the vessel.

The only conclusion to be drawn from what has preceded is, that putrefaction goes on with extreme slowness in cesspools, and only at the surface where the night soil is exposed to the atmosphere. If putrefactive change were active throughout the mass, the night soil would rapidly undergo changes in appearance, whereas in cesspools it retains its peculiar colour, consistence, and other qualities, for an indefinite period, the alteration being scarcely perceptible. If putrefaction, attended, as it would be, by the abundant extrication of gases, really occurred in cesspools, the bubbles of gas imprisoned in the viscid mass would cause the whole to swell to many times its original bulk, and to run over the sides of the cesspool, instead of lying as it does perfectly quiescent, and becoming so solid by subsidence at the bottom of old cesspools as to be capable of being dug out by the spade! Such is the condition of the contents of cesspools when the matter has lain at rest for a considerable number of years, and I may be permitted to remark, that it ill accords with the statements of many professed sanitarians, who have been the leaders of public opinion on the subject. All that I desire in making these remarks is, that a thorough examination should be made of the entire question, and that so large a quantity of most valuable matter should be employed, as it ought to be, in fertilising our fields.

A mistaken notion has also prevailed as to the nature of the fœtid emanations from cesspools of fæcal matters. For a long period the odour was attributed to the extrication of sulphuretted hydrogen. This is altogether a mistake, as the quantity of sulphuretted hydrogen produced by the putrefaction is very small, and after the whole has been separated by distillation with a weak alkaline solution, the distilled liquid retains the full odour of the night soil, and is ammoniacal. But even after the ammonia has been removed by a second distillation with dilute and sulphuric acid, the odour, although somewhat weaker, still persists, and is proved to be dependent on a volatile animal matter, which yields a rose red colour with concentrated nitric acid, which is changed to lemon yellow by supersaturation with ammonia. This substance I have proved to exist in smaller proportion in fresh fæces, immediately after their evacuation; but I have not been successful in isolating it, so as to study its properties. It contains nitrogen, however; for the distilled fluid freed from ammonia, on standing in a warm place for a few days, becomes distinctly ammoniacal.

The question now arises:—To what practical results do the preceding observations tend? It was absolutely hopeless, a few months since, to turn the public mind from the idea that cesspools, where well constructed and properly inspected, are not the "abominations" they have been termed; but the events of the few preceding weeks have so altered the circumstances of the case that I have been emboldened, at the risk of much misrepresentation, and perhaps abuse, to place my observations, and the conclusions I shall presently draw from them, before the public; and I shall feel perfectly satisfied if I succeed in drawing public attention to them, and causing a thorough scientific investigation of the entire subject.

I apprehend that it is now a settled point that the solid fæcal matters must not be permitted to enter our magnificent river and pollute its waters and its shores. But the real difficulty is to know what to do with the fæcal matters of two millions and a-half of persons congregated in the metropolis, after intermixture with millions of gallons of water. It may be said, We will excavate reservoirs, in which to allow the subsidence of the solid matter, and cast the liquid into the Thames. This would not be objectionable as regards the river; but objections of another kind are insuperable. The reservoirs must be of such enormous size as to require vast sums to be expended on their construction, because the fæcal matter, thoroughly mixed, as it would be, with the water, is somewhat slow in subsiding; and again, the entire soluble salts and other soluble matters of the soil would be carried away, leaving a substance of inferior value as a manure. The only other course, supposing it be determined to carry off the whole of the fæcal matters by sewers from London, is to carry them direct to the sea; but this plan is open to the most serious objections; viz., the enormous cost of the works, the small amount of fall to be obtained (unless by costly pumping machinery), which would allow of subsidence, the continual labour involved in cleaning the sewers, and the direct loss to the nation of what cannot be directly valued at less than three or four hundred thousand pounds, and is of incalculable indirect value in the additional crops produced by it as a manure. Here we have insuperable difficulties on all sides to the schemes for carrying off the fæcal matters on the gigantic sewerage scheme; and that scheme must be abandoned, or, after expenditure of enormous sums of money, and burdening the inhabitants of the metropolis with enormously increased rates for many years to come, entire failure will be the result. I do not hesitate to assert, and I am ready to prove by demonstration, that no necessity for any such large expenditure exists, if the erroneous notions now prevalent concerning cesspools be abandoned, and these are well constructed and placed under careful supervision. If putrefaction, as I have shewn, does not go on to any extent in properly constructed cesspools, it is clear that the injurious consequences attributed to them must be illusory, since only a very small quantity of mephitic gases can be extricated—far less than what will be extricated from the contents of sewers when all the fæcal matters are poured into them. A well constructed cesspool is easily made, at no great expense. A pit should be dug, sufficiently large to receive the fæcal matters of the inhabitants of the house or houses during five or six years. The walls should be made of bricks, built in with Roman cement, and puddled externally with clay when in a gravelly or other soil. The top must be closely covered, and a pan with a six-inch pipe should pass down some distance below the escape pipe drain, and in all cases water should be laid on. It is evident that, with such a construction, percolation through the surrounding soil would be impossible, even in the new cesspool; but after it had been once filled, the lining of viscid matter of the night soil would be a still further bar to percolation; and as the entire cesspool would be closed, except at the entrance of the drain pipe, little fresh atmospheric air could find its way into the upper part of the cesspool, and this would still further diminish putrefactive changes. The only nuisance would be, that once in five or six years the emptying of the cesspool would cause a bad odour in the house; but I apprehend that few would be hardy enough to assert that this would exercise any perceptible amount of detrimental influence on the health of the inhabitants.

No one can doubt that when cesspools are badly constructed, allowing percolation through the surrounding soil, or overflowing, or when they are not properly covered, they are great nuisances, and injurious to the health of the inhabitants of the houses to which they are attached; but the false argument has prevailed that, because they have been abused, they ought not to be used, and should be done away with as speedily as possible. The same argument would prevail against the use of beer, mutton, or wine, because some imprudent sensual persons overgorge themselves or become drunkards!

If, however, cesspools were established throughout London, on the construction I have described; and if at the same time they were placed under a strict supervision by proper officers appointed for the purpose, I am quite certain that no complaints, and no nuisance injurious to the public health, would ensue.

The next question to be discussed is, What shall we do with the liquid drainage? Shall we undertake enormous works for carrying it into the river at a distance from the metropolis, or may it be at once discharged into the river by the existing sewers, without detriment to the public health? This is a question of immense importance in an economical as well as a sanitary point of view. In answer, I would first remark that, until within the last twelve years, before the "barbarous" system was brought into force on a large scale, of turning the contents of cesspools into the common sewers, notwithstanding the constant flows of house drainage into the river, the water was comparatively pure; but, year by year, as that pernicious practice has been more extended, the river has become more and more foul, until public attention has at last been forcibly drawn to its condition, and the press has imperatively declared that the Thames must be purified.

As we no longer draw water for drinking from the Thames nearer than Kew, it is not necessary, nor indeed is it possible, to render the water absolutely pure and limpid. But it may be rendered sufficiently pure and inoffensive for all practical purposes, by simply preventing the solid excreta from entering its stream; the liquid portion being disposed of with the greatest possible rapidity by the natural process I shall now proceed to describe.

In a series of comparative analyses, undertaken by order of the Government, in the year 1851, by Professors Graham, Miller, and Hofmann, of the water supplies of the metropolis, we find the following curious results:—The number of grains of soluble organic matter in each gallon of Thames water, taken at the following places, was: at Thames Ditton, 2·29; at Kew, 3·07; at Barnes, 2·75; at Battersea (Chelsea Company), 2·38; at Battersea (Southwark and Vauxhall Company), 1·51; and at Lambeth, 2·59. Further, the organic matter of the water taken at Kew was found to give 0·105 of a grain of nitrogen, and that of the Southwark and Vauxhall Company, taken at Battersea, only 0·031 grain, shewing a very considerable progressive diminution as we descend the river to this point, not only in the quantity of organic matter, but also in the quantity of nitrogen it contained. But if we turn to another table, on page 6 of the Report, we find that while the other waters contain only traces of nitric acid and ammonia, the former has risen to 0·236 and the latter to 0·0309 in the Southwark and Vauxhall water; for while this water contained the smallest quantity of soluble organic matter, it was substituted by nitrate of ammonia; so that in reality, so far as relates to nitrogenised organic matter, the water taken at Battersea was more pure than either of the other waters drawn from the Thames. On the other hand, the suspended organic matter increases as we descend the Thames; but, as the Report relates only to the drinking water supplied by the companies, its amount would, of course, depend on the care expended on its filtration. The results were: water taken at Thames Ditton, 0·0; at Kew, 0·01; at Barnes, 0·02; at Chelsea, 0·0; at Battersea Fields, 1·92; and at Lambeth, 1·15. The irregularity of these figures demonstrates the interference of pollution on the results; as we cannot suppose that the suspended organic matter should be smaller in quantity at Lambeth than at Battersea; or that, in the natural condition of the water, it should be totally absent at Chelsea.

Another series of analyses, made by Mr. Aikin and Dr. A. S. Taylor, yielded the following results:—The organic matter in Thames water taken at Henley-on-Thames was 3·73 grains per gallon; at Caversham, 2·7 grains; at Kew, 2·13 grains; at

Chelsea, 1·37; at the Tower, taken at high water, 8·0 grains; showing a sudden and very large increase immediately below London. (*Minutes of Evidence, Metropolis Water Bill,* 1851.)

The course of these curious and somewhat startling results, obtained incidentally, in the course of an investigation undertaken for an entirely different purpose, and therefore more worthy of credit, is the decomposing action of aerated water on organic matter, whereby the organic matter is rapidly reduced to the ultimate products of its decomposition, carbonic acid, nitric acid, and ammonia. From the foregoing data, it will be seen that the quantity of soluble organic matter in the water taken near the Red House, Battersea Fields (Southwark and Vauxhall Company), was really only half the amount contained in the Kew water, and considerably less than that in the water at Thames Ditton; and that even the water taken at Lambeth contained less than that at Kew, and very little more than the water from Thames Ditton. How are these very curious results to be explained, except on the well known facts that organic matters, in contact and agitated with aerated water, are rapidly oxidised, and reduced to inorganic matter? The large proportion of nitric acid, compared with that of ammonia, in the Southwark and Vauxhall Company's water, taken in Battersea Fields, is interesting, because, had the proportions been reversed, the presence of ammonia might have been attributed to the numerous gas factories, whose gas-water, containing large quantities of hydrosulphate of ammonia, is discharged into the Thames; and it is possible that some part of the ammonia might be derived from that source.

However this may be, it is quite clear that, notwithstanding the constant drain of organic matter into the Thames at London, and the thorough intermixture of these matters with the water by the flow and ebb of the tide, the quantity of soluble nitrogenised matter is less near London than at Kew, and little more than at Thames Ditton. But, on the other hand, the quantity of suspended organic matter is much greater near London than higher up the stream, as we may readily convince ourselves by merely looking at the water. The soluble organic matter rapidly undergoes change; but the insoluble matter is much more slowly destroyed, because it is necessary that it should undergo a series of changes, before it can be brought into solution, and rendered amenable to the action of the aerated water. More than this, it is liable to be deposited with the mud on the strand of the Thames, and thus protected from the decomposing action of the air and water.

These views, so far as the destruction of organic matter is concerned, are supported by the evidence of Dr. A. S. Taylor and Dr. Clark, given before the committee on the Metropolitan Water Bill (*Minutes of Evidence*, p. 687). Dr. Taylor says, for instance, in answer to a question relative to the influence of the sewerage on the water: "All such substances are very rapidly decomposed and destroyed; the nitrogen is converted into nitric acid (and ammonia?); the sulphur is converted into sulphuric acid; so that those fœtid substances which go into the Thames from London, when rolled about by the action of the water containing an enormous amount of air, are all oxidised and destroyed. Within a certain limit they may be found; but still, after a very short passage, they are very soon, indeed, destroyed." And again, "with water not exposed to the air, and not containing air, it is most offensive and unwholesome; but with the fluid sewerage mixed with a comparatively small portion of water containing air, like the Thames, and exposing an enormous surface to the air in its daily motion, the effect is to obliterate every trace that a chemist can detect." Dr. Taylor further estimates that "by the time that the water has passed six or eight miles, according to wind or other circumstances, you may have a complete decomposition of it," *i.e.*, the offensive matter. There can be no doubt then that the Thames possesses, up to a certain point, the power of self-purification; but it must be remembered that, until within the last few years only insoluble matter found its way into the river. More recently, the plan of emptying cesspools into the drains having been carried on very extensively, the Thames has necessarily become more foul and offensive; and if this system be continued and extended, the purifying power will be overbalanced, and any part of the Thames into which the whole fæcal and other refuse matters may be discharged will become positively pestiferous.

In the foregoing observations, I have endeavoured, and I believe successfully, to prove two chief propositions:—

1. That cesspools, properly constructed, with pans and discharge pipe, are not injurious, because little if any putrefactive fermentation goes on in them, compared with that which must occur in sewers, into which fæcal matters are discharged.
2. That the liquid and soluble organic matters of sewer water are rapidly destroyed by the oxidising agency of the aerated water of the Thames, and may be discharged at once into the river, without danger to the public health.
3. That, consequently, by reverting to a proper system of cesspools, under supervision by officers appointed for the purpose, and continuing the present system of drainage, large sums of public money will be saved, the sewers will not be converted into "elongated cesspools;" the river will be practically pure for all purposes except drinking water; enormous public burdens in the shape of rates for useless, perhaps injurious, works will be avoided; and last, but not least, the material for increasing the fertility of the land will be preserved.

In conclusion, I may remark that the ultimate disposal of the contents of the cesspools may be so managed, by proper arrangements, as to be comparatively inoffensive, and at the same time innocuous, so far as regards the public health.

Wandsworth Road, Aug. 11th, 1856.

